# Quality of Life and Clinical Outcome After Traumatic Spleen Injury (SPLENIQ Study): Protocol for an Observational Retrospective and Prospective Cohort Study

**DOI:** 10.2196/12391

**Published:** 2019-05-06

**Authors:** Claudia PAM Raaijmakers, Paul NM Lohle, Paul Lodder, Jolanda de Vries

**Affiliations:** 1 ETZ Hospital (Elisabeth-TweeSteden Ziekenhuis) Trauma TopCare Tilburg Netherlands; 2 ETZ Hospital (Elisabeth-TweeSteden Ziekenhuis) Department of Radiology Tilburg Netherlands; 3 Tilburg University Department of Medical and Clinical Psychology Tilburg Netherlands; 4 Tilburg University Department of Methodology and Statistics Tilburg Netherlands; 5 ETZ Hospital (Elisabeth-TweeSteden Ziekenhuis) Department of Medical Psychology Tilburg Netherlands

**Keywords:** quality of life, QOL, spleen, trauma, injury, splenic artery embolization, SAE, embolization, magnetic resonance imaging, observational study

## Abstract

**Background:**

Little is known about the effect of a splenic rupture on the quality of life (QOL) of patients, although the spleen is one of the most frequently injured organs in blunt abdominal trauma. It is essential to obtain more knowledge about QOL after traumatic spleen injury so that this can be taken into account when choosing treatment.

**Objective:**

The primary objective of the SPLENic Injury and Quality of life (SPLENIQ) study is to determine QOL after treatment for traumatic spleen injury. The secondary objective is to investigate clinical and imaging outcome in relation to QOL.

**Methods:**

A combination of a retrospective single-center and a prospective multicenter observational cohort study will be conducted. Patients in the retrospective study have had a splenic injury after blunt abdominal trauma and were admitted for treatment to the ETZ Hospital (Elisabeth-TweeSteden Ziekenhuis) in Tilburg between January 2005 and February 2017. Concerning the prospective cohort study, patients with splenic injury admitted to 1 of the 10 participating hospitals between March 2017 and December 2018 will be asked to participate. The follow-up period will be 1 year regarding QOL, clinical symptoms, and imaging. Patients in the retrospective study will complete 2 questionnaires: World Health Organization QOL assessment instrument-Bref (WHOQOL-Bref) and 12-Item Short-Form Health Survey (SF-12). Patients in the prospective study will complete 5 questionnaires at 1 week, 1 month, 3 months, 6 months, and 12 months after treatment: WHOQOL-Bref, SF-12, Euroqol 5-Dimensional 5-Level (EQ-5D-5L) questionnaire, Institute for Medical Technology Assessment (iMTA) Productivity Cost Questionnaire (iPCQ), and iMTA Medical Consumption Questionnaire (iMCQ). In both the retrospective and prospective study, patients treated with splenic artery embolization will undergo magnetic resonance imaging (MRI). The retrospective group will undergo MRI once, and the prospective group will undergo MRI 1 month and 1 year after treatment. Treatment of splenic injury depends on the severity of the splenic injury, the hemodynamic condition of the patient, and the hospital’s or doctor’s preference. This study is observational in nature without randomization. Concerning the retrospective data, multivariate analysis of covariance will be done. With regard to the prospective data, mixed linear modeling will be performed.

**Results:**

This project was funded in April 2015 by ZonMw. The results of the retrospective study will be expected in March 2019. With regard to the prospective study, inclusion of patients was completed in December 2018 and data collection will be completed in December 2019. The first results will be expected in 2019.

**Conclusions:**

To our knowledge, this is the first study that examines QOL in patients with a traumatic spleen injury. The SPLENIQ study responds to the shortage of information about QOL after treatment for traumatic spleen injury and may result in the development of a patient-oriented protocol.

**Trial Registration:**

ClinicalTrials.gov NCT03099798; https://clinicaltrials.gov/ct2/show/NCT03099798 (Archived by WebCite at http://www.webcitation.org/714ZKV6A0).

**International Registered Report Identifier (IRRID):**

DERR1-10.2196/12391

## Introduction

### Background

Morbidity and mortality are the most commonly used outcome parameters in trauma care literature. However, most patients survive their trauma, and depending on the severity of the trauma, they will be limited in daily life, both physically and mentally. Although quality of life (QOL) is recognized as an important outcome measure, it is still a highly neglected aspect in trauma care studies, including studies with splenic injury patients. Multiple studies that did measure QOL have shown that severely injured patients suffer from psychological complaints and decreased QOL [1-3]. These factors have a major social and economic impact because trauma often involves young patients who frequently are unable to return to work, to reintegrate back into society, or to retrieve their previous activity level [4-11]. In case of splenic injury, treatment choices have to be made in which it is currently unknown what the effects for these patients will be in the short, medium, and long term. When more knowledge becomes available about QOL, it will be useful to determine the choice of treatment.

In blunt abdominal trauma, where the spleen is one of the most frequently injured organs, much is known about morbidity and mortality [12]. Internal bleeding caused by abdominal organ injury is one of the main causes of death after trauma, and a missed splenic rupture is the most common cause of preventable death in trauma patients [13,14]. Presently, the standard of care in hemodynamically stable patients is nonoperative management (NOM), involving close observation of the patient, with success rates up to 90% [12]. A recent study among adults with blunt splenic injury suggests that there are prognostic factors for failure of NOM. Strong evidence exists for prognostic patient factors such as age of 40 years or above, Injury Severity Score of at least 25, and American Association for the Surgery of Trauma splenic injury grade of 3 or higher [15]. Failure of therapy leads to more interventions, longer hospital stay, and higher mortality rates, resulting in increased costs and presumably decreased QOL. When NOM fails, angiography and splenic artery embolization (SAE) can be used as a supplement to NOM. The success rate of SAE ranges between 73% and 100%, with an overall success rate of NOM combined with SAE ranging between 86% and 100% (most studies reporting success rates greater than 90%) [16]. Despite this success rate, much remains unknown about splenic function after SAE, although it is speculated that there is a relationship between splenic volume and the immunologic status of the patient [17-20]. Preservation of splenic function might be one of the biggest advantages of NOM and SAE. However, patients treated with SAE have a risk of developing splenic infarction, abscesses, or cysts, with distal embolization having a significantly higher association with major complications compared with proximal embolization [21,22]. Surgery is indicated when a patient is hemodynamically unstable and does not respond to transfusion or when associated intraabdominal injuries require surgical management. Possible disadvantages of surgery are postsplenectomy complications, such as sepsis, thrombocytosis, and a lifetime risk of invasive infections (overwhelming postsplenectomy infection) [23,24]. All complications may have a major impact on patients’ QOL.

Cost-effectiveness is important in the choice of treatment. Published study results on hemodynamically stable patients with splenic injury favored nonsurgical management over surgery with better clinical and cost results. SAE as a supplement to NOM trended toward being more cost-effective with a shorter hospital stay, despite comparable failure rates. Procedure-related costs were higher for surgery than for SAE, but total hospital costs were not significantly different [25].

### Objectives

There is a growing demand for a (national) guideline or protocol for clinical decision making in traumatic spleen injury. Therefore, it is important to determine the optimal selection criteria for the appropriate management strategy. To achieve this, the entire process surrounding a patient with splenic injury must be considered. Even today, it remains unclear what the impact of QOL is on the entire process. Therefore, the primary objective of this project is to examine the QOL of patients after therapy (NOM, SAE, and surgery) for traumatic spleen injury using a retrospective and prospective group of patients. The secondary objective is to investigate the clinical outcome (eg, complications, reinterventions, and additional therapy), imaging outcome (diagnosis and magnetic resonance spleen imaging after SAE), and cost outcome (cost-effectiveness) and their relation to QOL.

Finally, the data and results acquired from this study may result in the development of a patient-oriented protocol for the management of traumatic spleen injury.

## Methods

### Study Design

A combination of a retrospective single-center and a prospective multicenter observational cohort study will be conducted, assessing the effects of NOM, SAE, and splenectomy in patients with splenic injury after blunt abdominal trauma.

### Participants and Centers of Recruitment

#### Retrospective Study

The study population comprises patients who had a splenic injury after blunt abdominal trauma and were admitted for treatment in the ETZ Hospital (Elisabeth-TweeSteden Ziekenhuis) in Tilburg, the Netherlands, between January 2005 and February 2017. It concerns both men and women who were 18 years or older at the time of screening (February 2017).

#### Prospective Study

The prospective study will be performed in 10 selected Dutch hospitals containing experienced interventional radiologists and trauma surgeons qualified to perform SAE and splenectomy, respectively. The group includes 7 level-1 trauma centers (Erasmus MC, ETZ Hospital, Leiden University Medical Center, Amsterdam University Medical Center, Radboud University Medical Center, Hospital Medisch Spectrum Twente, and Isala Hospital) and 3 level-2 trauma centers (Maasstad Hospital Rotterdam, Albert Schweitzer Hospital Dordrecht, and Amphia Hospital Breda). The study will be organized in a network infrastructure in which radiologists and trauma surgeons collaborate.

All patients with a splenic injury after abdominal trauma confirmed by ultrasound/focused assessment with sonography in trauma (US/FAST) and/or computed tomography (CT) at the primary trauma screening at the 10 participating hospitals between March 2017 and December 2018 will be asked to participate.

### Inclusion and Exclusion Criteria

#### Retrospective Study

The inclusion criteria are patients (1) diagnosed with splenic injury after trauma; (2) who underwent NOM, SAE, or surgery at the ETZ Hospital; (3) diagnosed between January 2005 and February 2017; and (4) who were aged 18 years or older at the time of screening (February 2017). Patients will be excluded from questionnaires in case of (1) insufficient knowledge of the Dutch language (verbal and writing) or (2) obviously, death. Patients treated with SAE will be excluded for magnetic resonance imaging (MRI) when they do not want to or are not able to undergo an MRI abdomen (eg, pregnancy or other contraindications).

#### Prospective Study

To be eligible to participate, patients (1) must be aged 18 years or older, (2) have splenic injury after abdominal trauma (confirmed by US/FAST and/or CT), and (3) must be treated in 1 of the 10 participating hospitals between March 2017 and December 2018. Exclusion criteria are identical to those of the retrospective study.

### Sample Size Calculation

As the World Health Organization QOL assessment instrument-Bref (WHOQOL-Bref) comprises multiple facets, the *retrospective data* will be analyzed using a multivariate analysis of covariance (MANCOVA). This technique is more powerful than a univariate analysis of variance, resulting in a required sample size of 135 patients giving a medium effect and power of 0.80. Previous research [26] indicates that the minimal clinically important difference (MCID) of the WHOQOL-Bref slightly varies across its domains. Table 1 shows for each domain the MCID and standard deviation. On the basis of these statistics, the Cohen *d* effect sizes were calculated and transformed via Cohen *f* to the *f*^2^ effect sizes required in the MANCOVA power analysis. It turns out that 138 participants are required to detect the average *f*^2^ of 0.061 with a power of 0.80, given a significance level of .05.

With regard to the *prospective data*, mixed linear modeling will be used. Power analysis is performed for a repeated measures design investigating the interaction between treatment and time. We assumed a significance level of .05 and a medium effect size of partial eta squared of 0.05 and an average correlation of 0.50 among the 5 repeated measurements. On the basis of this, we require 33 participants to test with a power of 0.80, whether the 3 treatment groups differ in their change in QOL over the follow-up time. When assuming an effect size similar to the MCID’s reported in the table above, 27 participants are required to detect the medium effect of *f*=0.248 with a power of 0.80.

### Study Procedures

#### Retrospective Study

The clinical and imaging data of all splenic injury patients will be collected from the electronic patient files and registered anonymously. To measure QOL, all patients who are still alive will receive a written letter explaining the study, an informed consent form, the questionnaires, and a prestamped return envelope by mail. When a patient is willing to participate, he/she will sign the consent form and send it back in the return envelope, together with the completed questionnaires, assessing QOL and health status. When patients do not complete or return the questionnaires within 2 weeks, they receive a phone call as a reminder. When patients do not want to participate, the reason will be noted if the patient wants to let it be known. The patients who underwent SAE will be called for a voluntary single MRI abdomen at the ETZ Hospital in Tilburg.

This study has been reviewed and approved by the Medical Ethical Committee Brabant (METC Brabant, protocol number: NL54339.028.15) on January 27, 2016. The study has also been approved by the local ethical committee of the ETZ Hospital on February 9, 2016.

**Table 1:** Minimal clinically important difference (MCID) per WHOQOL-Bref domain.

Domain	MCID	SD	Cohen *d*	Cohen *f*	Effect size *f*^2^
Physical	1.545	3.1	0.498	0.249	0.062
Psychological	1.259	2.5	0.504	0.252	0.063
Social	1.274	2.6	0.490	0.245	0.060
Environmental	1.142	2.3	0.497	0.248	0.062
General	0.876	1.8	0.487	0.243	0.059
Average	1.219	2.460	0.495	0.248	0.061

#### Prospective Study

In each participating hospital, an interventional radiologist and a trauma surgeon will be designated as principal investigators. Each hospital will also have a research assistant, most likely a radiology technologist or a member of the research team. The daily work will be carried out by the research assistant under the supervision of the principal investigator at the ETZ Hospital (CR). The research assistant will check daily whether potential eligible patients were admitted to the hospital. This will be done by checking the subscription list, verbally checking with the attending (resident) radiologist and trauma surgeon, and verifying the data in the electronic patient record. The subscription list will be placed at the dictation station of the radiologist, where the trauma diagnoses are reported. If a patient is treated for a traumatic spleen injury, baseline characteristics will be collected and he/she will be screened for the inclusion and exclusion criteria. The clinical data of all eligible patients will be collected anonymously in the database. As soon as the patient can talk and is lucid, the assistant will visit the patient to provide a verbal and written explanation about the study. The time for consideration of participation is 1 week. When a patient is willing to participate, he/she will sign a consent form. If not, the reason will be noted if the patient wants to let it be known. Each inclusion will be reported to the principal investigator (CR). Total follow-up time is 1 year after treatment with time points at 1 week and 1, 3, 6, and 12 months after treatment.

Patients will complete the questionnaires at all time points. The questionnaires of time point 1 will most likely be handed out to the patients at the hospital ward (intensive care, medium care, or general ward), usually by the research assistant of that hospital. At the other time points, patients will be sent an email to complete the questionnaires, assessing QOL, quality of care, health care consumption, and return back to work (if applicable). These questionnaires will be completed by the patient using a secure Web-based program: Data Management by Research Manager [27]. If a patient does not have internet, the questionnaires will be provided on paper with a prestamped return envelope. When patients do not complete or return the questionnaires within 2 weeks, they will receive a phone call as reminder. When patients no longer want to participate, the reason will be noted if they want to let it be known. Patients treated with SAE will receive an MRI abdomen 1 month and 1 year after treatment.

This study has been reviewed and approved by METC Brabant (protocol number: prospective study NL54542.028.16) on May 18, 2016. The study has also been approved by the local ethical committees of the ETZ Hospital (6-13-2016), Maasstad Hospital (7-28-2016), Erasmus MC (9-21-2016), Albert Schweitzer Hospital (10-31-2016), Amphia Hospital (2-23-2017), Leiden University Medical Center (10-17-2017), Amsterdam University Medical Center (VUmc) (1-11-2018), Hospital Medisch Spectrum Twente (1-25-2018), Radboud University Medical Center (5-3-2018), and Isala Hospital (5-17-2018).

### Data Collection

Data of the retrospective and prospective study will be recorded in a secure online database (Data Management by Research Manager, Health Solutions Deventer) [27]. In the prospective study, the database will be available to every research assistant. The program complies with the new legislation for collecting and processing personal data in medical scientific research: General Data Protection Regulation dated May 25, 2018.

#### Clinical Data

For both the retrospective and prospective study, data will be collected from the electronic patient records and trauma registry (Network Emergency Care Brabant). The trauma registry compiles prehospital and hospital data of all trauma patients admitted after presentation to the emergency department. Patients with splenic injury will be identified by the International Statistical Classification of Disease and Related Health Problems (ICD-10) and Abbreviated Injury Scale (AIS) diagnosis codes starting with S36.x and 5442, respectively.

The collected data concern the following: age, sex, systolic blood pressure, hemoglobin, Glasgow coma scale (at arrival), intubation (Yes/No), imaging (US and/or CT), grading spleen injury (American Association for the Surgery of Trauma; see Table 2)[28], type of treatment (NOM/SAE/splenectomy), complications, hospital stay (days), spleen in situ at discharge (Y/N), reinterventions (Y/N), rehospitalization (Y/N), and mortality.

**Table 2:** American Association for the Surgery of Trauma spleen injury scaling (1994 Revision).

Grade^a^		Injury description
**I**		
	Hematoma	Subcapsular <10% of surface area
	Laceration	Capsular tear <1 cm parenchymal depth
**II**		
	Hematoma	Subcapsular 10%-50% of surface area; or intraparenchymal <5 cm in diameter
	Laceration	1-3 cm parenchymal depth, which does not involve a trabecular vessel
**III**		
	Hematoma	Subcapsular >50% of surface area or expanding; or ruptured subcapsular or parenchymal hematoma; or intraparenchymal hematoma >5 cm or expanding
	Laceration	>3 cm parenchymal depth or involving trabecular vessels
**IV**	
	Laceration	Involving segmental or hilar vessels producing major devascularization (>25% of the spleen)
**V**		
	Laceration	Completely shattered spleen
	Vascular	Hilar vascular injury with devascularized spleen

^a^Advance one grade for multiple injuries, up to grade III.

#### Questionnaires

Patients in the retrospective study will complete the questionnaires once. Patients in the prospective study will complete questionnaires at 1 week and 1, 3, 6, and 12 months after treatment (see Table 3).

##### World Health Organization Quality of Life Assessment Instrument-Bref

QOL will be assessed with the World Health Organization Quality of Life Assessment Instrument-Bref (WHOQOL-Bref) [29]. This 26-item questionnaire is a short version of the WHOQOL-100, and it assesses 4 domains (physical health, psychological health, social relationships, and environment) as well as 1 general facet, *Overall QOL and General Health*. The questions in the domains are derived from the 24 facets of the WHOQOL-100, with 1 item from each of the facets. Each item is rated on a 5-point rating scale. Higher scores indicate a better QOL [29,30]. The WHOQOL-Bref has good psychometric properties [30-33].

##### 12-Item Short-Form Health Survey

The 12-Item Short-Form Health Survey (SF-12) is a shorter version of the 36-Item Short-Form Health Survey (SF-36), which will be used for evaluating individual patients’ health status, researching the cost-effectiveness of a treatment, and monitoring and comparing disease burden. The SF-12 covers 8 domains: physical functioning, role limitations because of physical problems, bodily pain, general health, vitality, social functioning, role limitations because of emotional problems, and mental health [34,35]. From these domains, summary scores for the physical component (PCS) and mental component (MCS) can be computed. The 12 items for the SF-12 were selected such that the SF-12 component scores explain 90% of the variability in PCS and MCS scores of the SF-36 [34]. The SF-36 was used as a criterion for validation of the SF-12. The SF-12 and the SF-36 components and scales are scored with the algorithms specified by the developer [35]. The minimum possible score is 0 and the maximum possible score is 100. The SF-12 has good reliability and validity [36-42].

##### Euroqol 5-Dimensional 5-Level Questionnaire

The Euroqol 5-Dimensional 5-level questionnaire (EQ-5D-5L) is a generic health status instrument that measures health-related QOL [43]. The descriptive system of the instrument comprises 5 dimensions (mobility, self-care, usual activities, pain/discomfort, and anxiety/depression), which can be scored with 5 levels (ranging from no problems to severe problems). For the purpose of cost-effectiveness studies, health status is expressed in utilities, with a scale from 0 (death) to 1 (perfect health). The EQ-5D-5L can be used to derive utilities; the Dutch tariff can be used for this purpose [44]. Moreover, the EQ-5D-5L has good psychometric properties [45-50].

##### iMTA Productivity Cost Questionnaire

The impact of disease on the ability of a person to perform work should be part of an economic evaluation when a societal perspective is applied. The iMTA Productivity Cost Questionnaire (iPCQ) is a generic, nondisease-specific questionnaire, and it is applied in national and international studies [51]. The questionnaire is currently available in more than 10 languages, including Dutch. Both indirect cost because of absenteeism and the productivity losses because of presenteeism (ie, sick, but working) are taken into account. A manual is available, containing information on the modular structure of the iPCQ and its scoring and valuation methods that are used for cost calculations. By applying productivity costs, the answers of the iPCQ can be monetized and, as such, used in health economic evaluations.

**Table 3:** Timeline of the prospective study concerning measurements.

Time point	Timing and setting	Demographics	Questionnaires	Imaging
T1	After treatment, during admission	Demographic factors and clinical data	Quality of life (WHOQOL-Bref^a^); health status (SF-12^b^); and health-related QOL^c^ (EQ-5D-5L^d^)	—^e^
T2	1 month after treatment, at home (MRI^f^: outpatient clinic)	—^g^	Quality of life (WHOQOL-Bref); health status (SF-12); health-related QOL (EQ-5D-5L); productivity costs (iPCQ^h^); and medical consumption (iMCQ^i^)	MRI (SAE^j^ patients only)
T3	3 months after treatment, at home	—^g^	Quality of life (WHOQOL-Bref); health status (SF-12); health-related QOL (EQ-5D-5L); productivity costs (iPCQ); and medical consumption (iMCQ)	—^e^
T4	6 months after treatment, at home	—^g^	Quality of life (WHOQOL-Bref); health status (SF-12); health-related QOL (EQ-5D-5L); productivity costs (iPCQ); and medical consumption (iMCQ)	—^e^
T5	1 year after treatment, at home (MRI: outpatient clinic)	Clinical data	Quality of life (WHOQOL-Bref); health status (SF-12); health-related QOL (EQ-5D-5L); productivity costs (iPCQ); and medical consumption (iMCQ)	MRI (SAE patients only)

^a^WHOQOL-Bref: World Health Organization Quality of Life assessment instrument-Bref.

^b^SF-12: 12-Item Short-Form Health Survey.

^c^QOL: quality of life.

^d^EQ-5D-5L: Euroqol 5-Dimensional 5-Level questionnaire.

^e^At this time point, no MRIs have been completed.

^f^MRI: magnetic resonance imaging.

^g^At this time point, no demographic or clinical data have been collected.

^h^iPCQ: iMTA Productivity Cost Questionnaire.

^i^iMCQ: iMTA Medical Consumption Questionnaire.

^j^SAE: splenic artery embolization.

##### iMTA Medical Consumption Questionnaire

The iMTA Medical Consumption Questionnaire (iMCQ) is a generic, nondisease-specific instrument for measuring (direct) medical costs [52]. The instrument is a standardized self-reported questionnaire. The iMCQ includes questions related to frequently occurring contacts with health care providers and can be complemented with extra questions that are relevant for specific study populations. A manual is available for a structured use of the questionnaire. For the valuation of resource use, as obtained from the iMCQ, reference unit prices can be used. These reference prices can be derived from the Dutch manual for costing studies. The manual was commissioned and published by Zorginstituut Nederland and authored by the Institute for Medical Technology Assessment (iMTA) [53].

#### MRI Abdomen After Splenic Artery Embolization

Patients in the retrospective study will undergo MRI once at the ETZ Hospital in Tilburg, and patients in the prospective study will undergo MRI 1 month and 1 year after treatment at the hospital where the treatment took place. Not all hospitals have the same MRI scanner. However, the same scan protocol will be used at all locations, leading to comparable images and assessments (see Table 4).

Only patients treated with SAE will receive an MRI of the upper abdomen to evaluate the spleen morphologically (volume, necrosis, splenosis, calcifications, and chronic infarction) and dynamically (diffusion and enhancement).

**Table 4:** Magnetic resonance imaging scan protocol.

Retrospective study	Prospective study
Axial Dual FFE^a^/GRE^b^ (in-out phase); Coronal T2-weighted TSE^c^/FSE^d^; Axial T2-weighted TSE/FSE; Axial BFFE^e^/BGRE^f^ Volume (5 mm slices, no gap); Axial DWI^g^ with b value=0/400/800	Axial Dual FFE/GRE (in-out phase); Coronal T2-weighted TSE/FSE; Axial T2-weighted TSE/FSE; Axial BFFE/BGRE Volume (5 mm slices, no gap); Axial DWI with b value=0/400/800
3D^h^ (noncontrast)	Axial dynamic T1-weighted noncontrast with fat sat; Axial T1-weighted 3D magnetic resonance angiography contrast-enhanced; Axial dynamic T1-weighted contrast-enhanced (2 time points) with fat sat

^a^FFE: Fast Field Echo.

^a^GRE: Gradient Echo.

^c^TSE: Turbo Spin Echo.

^d^FSE: Fast Spin Echo.

^e^BFFE: Balanced Fast Field Echo.

^f^BGRE: Balanced Gradient Echo.

^g^DWI: Diffusion Weighted Images.

^h^3D: three-dimensional.

### Statistical Analysis

All analyses will be conducted using SPSS V24.0 (Statistical Package for Social Sciences, Chicago Illinois, USA). Frequencies and descriptive statistics will be calculated to provide an overview of the characteristics of the study population. Statistical test results will be considered significant at a level of *P*<.05.

Concerning the retrospective study, a MANCOVA will be done after correcting theoretically important covariates to assess the differences between treatment groups on the 4 WHOQOL-Bref domains and the general facet. For each type of treatment, a 1 sample *t* test will be performed for each WHOQOL-Bref scale to compare the QOL scores with reference data.

For both the retrospective and prospective studies, a logistic regression analysis will be performed on the outcome variables’ (1) need for reintervention (yes/no) and (2) for each complication (yes/no), assessing the effect of treatment after correcting for theoretically important covariates. With regard to hospital stay in days, a Kruskal-Wallis test will be performed with the group (type of treatment) and days in hospital. Analysis of covariance will be used to compare proximal versus distal SAE, thereby correcting the effect for theoretically important covariates. To confirm/find prognostic factors for failure of NOM, a logistic regression analysis will be performed if NOM is a failure quickly after treatment. Otherwise, a survival analysis will be performed.

Regarding the prospective study, to assess the differences between groups in their change in QOL over time, a linear mixed model analysis will be conducted. To answer our research question, we will focus on the interaction effect between measurement occasion and treatment group, while correcting for theoretically important covariates. For the repeated measures, an unstructured covariance matrix will be used. Item-level missing values will be imputed according to the guidelines of the questionnaires. Scale-level missing values will be handled directly through maximum likelihood estimation, because the mixed model procedure makes use of all available data for each participant over all time points.

To conduct the cost-effectiveness analysis, a cost-effectiveness model will be developed. This model will comprise 2 treatment arms; SAE will be compared with splenectomy. The cost-effectiveness study will be conducted according to the most recent Dutch guidelines for health economic research [54]. As such, the study will be performed from the societal perspective, which means that all costs and benefits should be considered, regardless of by whom the costs are borne or to whom the benefits accrue.

Effects will be expressed in quality-adjusted life years, which constitute a combination of QOL and length of life. QOL will be measured in utilities. Utilities express QOL on a scale from 0 (death) to 1 (perfect health). Utilities can be derived from the EQ-5D-5L [44]. Survival will be derived from international clinical literature. Costs will be estimated according to the Dutch Manual for Costing in Economic Evaluations, update 2015, using a societal perspective [54]. A bottom-up methodology will be used to compute costs; the total number of medical contacts will be multiplied with unit costs. Direct medical costs comprise all costs directly relating to the prevention, diagnostics, therapy, rehabilitation, and care of the intervention. Health care utilization will be derived from the iMCQ [52]. The iPCQ will be used to assess productivity losses [51]. The friction cost method will be used for the calculation of costs because of production losses [55]. This method is the expertise of the iMTA; it is its standard method and has been widely used.

### Regulation Statement

The study will be conducted according to the principles of the Declaration of Helsinki (7th revision, 64th World Medical Association General Assembly, Fortaleza, Brazil, October 2013) and in accordance with the Medical Research Involving Human Subjects Act (WMO).

## Results

For the retrospective study, the data collection has taken place and the database is complete. Results will be reported in March 2019. Enrollment of participants in the prospective study began in March 2017, and it was completed in December 2018. Data collection will be completed in December 2019. The first results will be reported in 2019.

## Discussion

The SPLENic Injury and Quality of life (SPLENIQ) study is the first study that examines the effect of traumatic spleen injury on patients’ QOL in both a retrospective and prospective observational study design. It also examines clinical and imaging outcome as well as cost-effectiveness. For several reasons, these studies add relevant information to the existing literature. First, there is a need for research into QOL after traumatic spleen injury, because this is an important but neglected factor in the trauma care literature. If more knowledge becomes available, this can be taken into account when choosing treatment. Second, the prospective study will be conducted in a multicenter context in 10 hospitals, involving a trauma surgeon and interventional radiologist in each hospital. This creates a strong collaboration between the participating hospitals and medical specialties, which hopefully adds to the inclusion rate. Third, it is still unknown what impact different SAE techniques and materials have on the morphology and volume of the spleen. To investigate this, using MRI is innovative and will provide interesting images containing important and necessary information.

Several factors related to the design and execution must be taken into account. First, patients will be treated with NOM, SAE, or surgery for a specific clinical condition. It may be the clinical situation that determines the long-term outcome, although that outcome is not or partially the result of the treatment. This risk is confounding by indication: the risk that the groups are in fact not easily comparable (ie, selection bias). To keep this to a minimum, the reason for choosing a particular treatment will be registered. In our analysis, we will correct for these confounding factors using propensity score analysis. Correction is only possible when adequate and good-quality information is available about the clinical condition of the patients, which led to the decision. The patient’s record will be searched thoroughly to find this information. Second, trauma patients often have multiple injuries that can affect QOL and clinical outcomes. We are not primarily interested in these additional injuries, but these will be included as a covariate in the analysis. Third, response bias may occur in the questionnaires group. Patients may decline participation because they are not interested or it may be too confronting to think/correspond about their psychological state. Fourth, the severely injured patients may be overrepresented in the nonresponse group concerning the questionnaires. To limit this, these patients will be visited as soon as they are approachable and, if necessary, will be provided assistance to complete the questionnaires. Fifth, the absence of randomization is a (strong) limitation and a potential source of bias, but randomized comparison in managing trauma patients is virtually impossible. Furthermore, it will have strong ethical implications as it is well known that randomizing trauma patients with intraabdominal bleeding, potentially unstable, is something not feasible in clinical practice. Sixth, the sample size calculation is based on the primary objective of the study. For the clinical and cost-effectiveness analysis, this implies that because of the small sample size, the uncertainty about the outcome is large. Seventh, we are aware of response shift bias. Response shift is a change in self-reported QOL that is a result of a change in internal standards (ie, recalibration), values (ie, reprioritization), or meaning of QOL (ie, reconceptualization) [56,57]. Thus, response shift reflects psychological adaptation: we do not consider this as a problem but as a fact of life. In addition, the method to prevent response shift contains recall bias itself. Considering this, and the fact that QOL is a generic outcome measure, ensures that we use the chosen method. Moreover, the retrospective patients will not be included in the prospective study.

In conclusion, the SPLENIQ study responds to the shortage of information about QOL after treatment for traumatic spleen injury. With developing a patient-oriented protocol, a necessary step is taken to customize standard care, which may contribute to a positive effect on QOL and clinical outcome.

## Abbreviations

CT: computed tomography
EQ-5D-5L: Euroqol 5-Dimensional 5-Level questionnaire
ETZ: Elisabeth-TweeSteden Ziekenhuis
FAST: focused assessment with sonography in trauma
iMCQ: iMTA Medical Consumption Questionnaire
iMTA: Institute for Medical Technology Assessment
iPCQ: iMTA Productivity Cost Questionnaire
MANCOVA: multivariate analysis of covariance
MCID: minimal clinically important difference
MCS: summary scores for the mental component (SF-12)
METC: Medical Ethical Committee
MRI: magnetic resonance imaging
NOM: nonoperative management
PCS: summary scores for the physical component (SF-12)
QOL: quality of life
SAE: splenic artery embolization
SF-12: 12-Item Short-Form Health Survey
SF-36: 36-Item Short-Form Health Survey
SPLENIQ: SPLENic Injury and Quality of life
US: ultrasound
WHOQOL-Bref: World Health Organization Quality of Life assessment instrument-Bref

## Appendix

Multimedia Appendix 1 Peer-reviewer report 1 from ZonMw.

Multimedia Appendix 2 Peer-reviewer report 2 from ZonMw.

Multimedia Appendix 3 Peer-reviewer report 3 from ZonMw.
