# Supplementary material for: Quality of Life and Clinical Outcome After Traumatic Spleen Injury (SPLENIQ Study): Protocol for an Observational Retrospective and Prospective Cohort Study
Source: JMIR Res Protoc. 2019 May 6;8(5):e12391. doi: 10.2196/12391 (PMC6533045; doi:10.2196/12391)
Supplement: Multimedia Appendix 1 [file resprot_v8i5e12391_app1.pdf]

Subsidieprogramma / Subsidy programme	:	**TopZorg**
Dossiernummer / Dossier number	:	**80-84200-98-15221**
Aanvrager / applicant	:	**Prof. dr. J.A. Roukema**
Projecttitel / Project title	:	**Traumatic Splenic Injury and Management**
Beoordelingscode / Assessment code	:	**B.2015.00250**

1. Information

Thank you, again, for finding the time to provide us with your expert opinion. Note that it is very important for us that you not only provide a score for the various criteria, but also elaborate on the strong as well as the weak(er) points for each criterion. We emphasize once more that the research proposal you are about to review is strictly confidential and needs to be treated accordingly.

Review process

The TopZorg committee will base its final quality assessment on the grant application itself, the reviewers' assessments and the applicant's rebuttal. Your assessment of the application will be sent to the applicant anonymously. The applicant will then have an opportunity to respond. The committee will also receive only anonymous versions of the received assessments. We would, therefore, strongly urge you to avoid any references to yourself in your reviewer's report.

If you accept this request to act as a referee for the specific application(s) referred to in this e-mail, ZonMw will assume that you can in fact be regarded as an independent expert in the field of the application as referred to in the ZonMw Conflict of Interest Code (http://www.zonmw.nl/en/about-zonmw/method/). If ZonMw is wrong in assuming this, ZonMw would be obliged if you would inform it as such - preferably forthwith - so that it can find another referee in good time. If you have any doubts regarding the Conflict of Interest Code, please feel free to contact us. For further information, please see the guideline under 'help' on the left side of the review form.

2. Quality criteria

Legenda: VG (Very good), G (Good), S (Sufficient), M (Moderate), U (Unsatisfactory)

2.1 Objective, problem definition and additional value to current knowledge

VG	G	S	M	U
X				

Consider:

- how clear and specific is the objective?;
- how clear and verifiable are the problem definition and hypothesis and is it consistent with the objective?;
- will this project yield new information?;
- ensure it does not duplicate past or ongoing projects.

Please indicate the strong and weak(er) points.

This is an excellent effort of investigating the potential advantages and differences among the currently available treatment options in treating Blunt Splenic injuries.
I also had recently the feeling of the need of such study in Trauma Surgery and the authors have to be commended in their purpose!
The strongest limitation of the study is the non-randomized nature of the study. The study is presented as a combination of a retrospective and a prospective multicentre cohort study.
I assume that in fact two different cohorts will be investigated and probably two studies can be derived. The retrospective one will be sort of a derivation cohort for assessing the outcomes and eventually identifying the prognostic factors predictors of NOM failure. The prospective cohort will otherwise represent a validation cohort for confirming prospectively the initial results.
The abscence of randomization is a strong limitation and a potential source of bias but we must admit that a randomized comparison in managing Trauma patients is virtually impossible and whenever feasible will have strong ethical implications. It is well known that randomizing trauma patients with intra-abdominal bleeding, potentially unstable, is something not feasible in clinical practice.
Nonetheless I feel the current study design is fairly acceptable.

Minor suggestions: Add within the Title "Traumatic Splenic Injury and Management" the detail that you are focusing on BSI (Blunt Injuries). This is clear in the methods and main text but I think it needs to be clear in the title too. Traumatic Splenic Injury and Management is still generic and might include all types of splenic injuries.

2.2 Strategy

VG	G	S	M	U
	X			

Consider:
- clarity;
- adequacy in terms of problem definition/assignment;
- adequacy of chosen method, study design and analyses;
- adequacy of outcome parameters and sample size (note that - considering the outcome parameters - the patient interest and/or health gain perspective should be taken into account);
- description of data collection and the protocol to be followed;
- the way in which the strategy reflects the factors gender, age, ethnicity and/or other characteristics relevant to the objective;
- degree of alignment/collaboration with intermediate and/or ultimate target group (the patient/client perspective);
- the way how possible data(sets) can and will be used and how data will be made available following completion of the project.

Please indicate the strong and weak(er) points.

There will be any case-mix adjustment for gender, age, AAST grade and other parameters?
Any subgroup analyses?
I would also suggest in the follow-up assessment, the incidence of OPSI or the occurrence of severe infectious complications in the group of patients underwent surgery and splenectomy.

I would like the authors to describe a little bit better the methods of assessing the AAST grade of the lesions. Do all patients will get a CT scan? Who is going to give a grade to each splenic lesion? A consultant Radiologist? There will be the possibility of re-review the CT scan-slides for a second opinion?
This is an important point to be clarified because the mis-classification of the AAST grading is a common mistake and a potential severe bias in interpreting the results of Splenic Trauma management

2.3 Knowledge transfer and implementation

VG	G	S	M	U
	X			

Consider:
- analysis of the context in which implementation is to take place;
- extent to which target groups are mentioned and involved;
- participation of stakeholders, other than target groups;
- prospect of structural incorporation in system;
- the plans for knowledge utilisation.

Please indicate the strong and weak(er) points.

The study includes patients treated at Level 1 and 2 Trauma centers. These are well known centers for high level of care for Trauma Patients.
Will the results be reproducible also in the suburban hospitals and Non Trauma Centers? Will the study provide a flow chart or an algorithm easy to use even in such hospitals? Or at least will be provided data on how to manage trauma patients admitted to suburban hospitals not having a full range of available tools (such as Trauma Surgeons on call, Interventional radiology 24/7, immediately accessible CT etc?)? Example what patients can be safely observed and sterted NOM and transferred the following morning to the Trauma Center for observation/ eventual AE? what subgroup of patients should NOT be transferred (e.g. unstable patients, signs of ongoing bleeding)

2.4 Project group and collaboration

VG	G	S	M	U
	X			

Consider:
- relevant expertise and disciplines;
- familiarity with research area;
- prior activities and products.
- collaboration with other institutions (note that within the TopZorg Programme collaboration with either a University Medical Center and/or scientific research institute is obliged)

Please indicate the strong and weak(er) points.

The group of investigators seems to be confident with the area of expertise (trauam surgery and management) and with the research methods

2.5 Feasibility

VG	G	S	M	U
X				

Consider:
- prospects of achieving the objective(s) using this strategy;
- realistic phasing and timetable;
- availability of facilities/staff;
- research protocol;
- realistic number of patients/institutes/organisations;
- recruitment of patients. If applicable: is randomisation feasible?

Please indicate the strong and weak(er) points.

I have already discussed the difficulty of randomization in the management of trauma patients. The overall schedule seems reasonable.

2.6 Overall quality assessment

VG	G	S	M	U
X				

Give your overall quality assessment regarding this grant application. Please indicate the most important strong and weak(er) points.

Overall is a worthwile study and something that may provide some evidence that the Trauma Surgery community definitely need

3. Budget

Legenda: TH (Too high), R (Realistic), TL (Too low), NJ (No judgement)

3.1 Budget

TH	R	TL	NJ
			X

Please give your judgement of the budget based on the data supplied in the grant application. There is a comprehensive overview of the requested budget (in Dutch).

If you are not able to assess the requested budget please type 'No Judgement' in the textbox.

Being from a foreign country I can not make a proper judgement on the budget but roughly seems reasonable for such study
