# Supplementary material for: Quality of Life and Clinical Outcome After Traumatic Spleen Injury (SPLENIQ Study): Protocol for an Observational Retrospective and Prospective Cohort Study
Source: JMIR Res Protoc. 2019 May 6;8(5):e12391. doi: 10.2196/12391 (PMC6533045; doi:10.2196/12391)
Supplement: Multimedia Appendix 2 [file resprot_v8i5e12391_app2.pdf]

Subsidieprogramma / Subsidy programme	:	**TopZorg**
Dossiernummer / Dossier number	:	**80-84200-98-15221**
Aanvrager / applicant	:	**Prof. dr. J.A. Roukema**
Projecttitel / Project title	:	**Traumatic Splenic Injury and Management**
Beoordelingscode / Assessment code	:	**B.2015.00251**

1. Information

Thank you, again, for finding the time to provide us with your expert opinion. Note that it is very important for us that you not only provide a score for the various criteria, but also elaborate on the strong as well as the weak(er) points for each criterion. We emphasize once more that the research proposal you are about to review is strictly confidential and needs to be treated accordingly.

Review process

The TopZorg committee will base its final quality assessment on the grant application itself, the reviewers' assessments and the applicant's rebuttal. Your assessment of the application will be sent to the applicant anonymously. The applicant will then have an opportunity to respond. The committee will also receive only anonymous versions of the received assessments. We would, therefore, strongly urge you to avoid any references to yourself in your reviewer's report.

If you accept this request to act as a referee for the specific application(s) referred to in this e-mail, ZonMw will assume that you can in fact be regarded as an independent expert in the field of the application as referred to in the ZonMw Conflict of Interest Code (http://www.zonmw.nl/en/about-zonmw/method/). If ZonMw is wrong in assuming this, ZonMw would be obliged if you would inform it as such - preferably forthwith - so that it can find another referee in good time. If you have any doubts regarding the Conflict of Interest Code, please feel free to contact us. For further information, please see the guideline under 'help' on the left side of the review form.

2. Quality criteria

Legenda: VG (Very good), G (Good), S (Sufficient), M (Moderate), U (Unsatisfactory)

2.1 Objective, problem definition and additional value to current knowledge

VG	G	S	M	U
X				

Consider:

- how clear and specific is the objective?;
- how clear and verifiable are the problem definition and hypothesis and is it consistent with the objective?;
- will this project yield new information?;
- ensure it does not duplicate past or ongoing projects.

Please indicate the strong and weak(er) points.

Hypotheses and objectives are clear albeit ambitious.
Primary Objective: Quality of life associated with blunt splenic trauma management strategies. This is certainly an area that is lacking in the literature and deserves attention.
Secondary Objectives: Determining complications and elucidating factors associated with failure of the treatment strategies are clear and appropriate areas of study. Assessment of splenic funtion based on MRI morphology is an interesting thought and should provide very interesting imaging however at best a surogate marker of splenic function. There is no currently accepted serologic or imaging marker of immunologic function of the spleen

2.2 Strategy

VG	G	S	M	U
	X			

Consider:

- clarity;
- adequacy in terms of problem definition/assignment;
- adequacy of chosen method, study design and analyses;

- adequacy of outcome parameters and sample size (note that - considering the outcome parameters - the patient interest and/or health gain perspective should be taken into account);
- description of data collection and the protocol to be followed;
- the way in which the strategy reflects the factors gender, age, ethnicity and/or other characteristics relevant to the objective;
- degree of alignment/collaboration with intermediate and/or ultimate target group (the patient/client perspective);
- the way how possible data(sets) can and will be used and how data will be made available following completion of the project.

Please indicate the strong and weak(er) points.

Sample size calcuation should at least in part be based on the hypothesized difference in the magnitude of your're primary outcome variable(s). This was not clear.
Exclusion criteria includes mortality ... while one cannot perform QOL assessment on patients who succumb mortality must be captured in order to determine utilities when computing QUALY's. Reporting mortality must be done.
A referenced statement that your QOL assessment tool has been validated in the trauma population is necessary.
On-line administration of the QOL assessment tool is never as good as a standardized face to face evaluation.
Further detail is required to risk adjust the primary outcome; for example, the patient's traumatic brain injury may have a much greater impact on the patient's QOL than the management strategy used to treat their blunt splenic trauma.

2.3 Knowledge transfer and implementation

VG	G	S	M	U
X				

Consider:

- analysis of the context in which implementation is to take place;
- extent to which target groups are mentioned and involved;
- participation of stakeholders, other than target groups;
- prospect of structural incorporation in system;
- the plans for knowledge utilisation.

Please indicate the strong and weak(er) points.

All areas well defined, described and feasible.

2.4 Project group and collaboration

VG	G	S	M	U
X				

Consider:

- relevant expertise and disciplines;
- familiarity with research area;
- prior activities and products.
- collaboration with other institutions (note that within the TopZorg Programme collaboration with either a University Medical Center and/or scientific research institute is obliged)

Please indicate the strong and weak(er) points.

Acknowledged experts in fields relevant to this research.
Collaboration seems established and appropriate.

2.5 Feasibility

VG	G	S	M	U
X				

Consider:

- prospects of achieving the objective(s) using this strategy;
- realistic phasing and timetable;
- availability of facilities/staff;
- research protocol;
- realistic number of patients/institutes/organisations;
- recruitment of patients. If applicable: is randomisation feasible?

Please indicate the strong and weak(er) points.

This will be an ambitous undertaking however the projected timetable seems appropriate. Sample size calculations need to be revisited based on earlier comments regarding hypothesized (and possibly the literature estimates) magnitude of the effect difference in your primary outcome measure.

2.6 Overall quality assessment

VG	G	S	M	U
X				

Give your overall quality assessment regarding this grant application. Please indicate the most important strong and weak(er) points.

A clearly well thought out and ariticuated project, an ambitious undertaking that will add to literature in splenic trauma management. My principle concern is undertaking too much, that is, limiting the work to a few less goals may ultimately result in stronger work.

3. Budget

Legenda: TH (Too high), R (Realistic), TL (Too low), NJ (No judgement)

3.1 Budget

TH	R	TL	NJ
			X

Please give your judgement of the budget based on the data supplied in the grant application. There is a comprehensive overview of the requested budget (in Dutch).

If you are not able to assess the requested budget please type 'No Judgement' in the textbox.

Have no expertise in this area
