# Supplementary material for: Quality of Life and Clinical Outcome After Traumatic Spleen Injury (SPLENIQ Study): Protocol for an Observational Retrospective and Prospective Cohort Study
Source: JMIR Res Protoc. 2019 May 6;8(5):e12391. doi: 10.2196/12391 (PMC6533045; doi:10.2196/12391)
Supplement: Multimedia Appendix 3 [file resprot_v8i5e12391_app3.pdf]

Subsidieprogramma / Subsidy programme	:	**TopZorg**
Dossiernummer / Dossier number	:	**80-84200-98-15221**
Aanvrager / applicant	:	**Prof. dr. J.A. Roukema**
Projecttitel / Project title	:	**Traumatic Splenic Injury and Management**
Beoordelingscode / Assessment code	:	**B.2015.00252**

1. Information

Thank you, again, for finding the time to provide us with your expert opinion. Note that it is very important for us that you not only provide a score for the various criteria, but also elaborate on the strong as well as the weak(er) points for each criterion. We emphasize once more that the research proposal you are about to review is strictly confidential and needs to be treated accordingly.

Review process

The TopZorg committee will base its final quality assessment on the grant application itself, the reviewers' assessments and the applicant's rebuttal. Your assessment of the application will be sent to the applicant anonymously. The applicant will then have an opportunity to respond. The committee will also receive only anonymous versions of the received assessments. We would, therefore, strongly urge you to avoid any references to yourself in your reviewer's report.

If you accept this request to act as a referee for the specific application(s) referred to in this e-mail, ZonMw will assume that you can in fact be regarded as an independent expert in the field of the application as referred to in the ZonMw Conflict of Interest Code (http://www.zonmw.nl/en/about-zonmw/method/). If ZonMw is wrong in assuming this, ZonMw would be obliged if you would inform it as such - preferably forthwith - so that it can find another referee in good time. If you have any doubts regarding the Conflict of Interest Code, please feel free to contact us. For further information, please see the guideline under 'help' on the left side of the review form.

2. Quality criteria

Legenda: VG (Very good), G (Good), S (Sufficient), M (Moderate), U (Unsatisfactory)

2.1 Objective, problem definition and additional value to current knowledge

VG	G	S	M	U
	X			

Consider:

- how clear and specific is the objective?;
- how clear and verifiable are the problem definition and hypothesis and is it consistent with the objective?;
- will this project yield new information?;
- ensure it does not duplicate past or ongoing projects.

Please indicate the strong and weak(er) points.

Zowel SAE als splenectomie zijn aanvaarde behandelingsopties voor milttrauma; echter analyse van de potentiele voordelen van de ene therapie over de andere op basis van een gerandomiseerde prospectieve studie is momenteel niet beschikbaar. Bijkomend lijkt het niet erg waarschijnlijk dat dit in de nabije toekomst internationaal wordt onderzocht, mede gezien de beperkte interesse van de industrie (goedkope devices zoals coils enz..)

2.2 Strategy

VG	G	S	M	U
		X		

Consider:

- clarity;
- adequacy in terms of problem definition/assignment;
- adequacy of chosen method, study design and analyses;
- adequacy of outcome parameters and sample size (note that - considering the outcome parameters - the patient interest and/or health gain perspective should be taken into account);
- description of data collection and the protocol to be followed;

- the way in which the strategy reflects the factors gender, age, ethnicity and/or other characteristics relevant to the objective;
- degree of alignment/collaboration with intermediate and/or ultimate target group (the patient/client perspective);
- the way how possible data(sets) can and will be used and how data will be made available following completion of the project.

Please indicate the strong and weak(er) points.

hier lijken er toch wel wat tekortkomingen te zijn. zo bv. is het me niet duidelijk hoe je in de eerste studie (retrospectieve studie) een QoL analyse zal uitvoeren.
Verder lijkt met me onduidelijk of elke patient met een milttrauma (ook deze met een graad V miltfractuur) potentieel kan gerandomiseerd worden nnar NOm of SAE.
Verder begrijp ik niet goed waarom patienten die sterven tijdens of onmiddellijk na de interventie geexcludeerd worden uit de studie; mede gezien de patienten voor de ingreep worden gerandomiseerd.
Tenslotte is het me ook niet duidelijk wat er gebeurt met patienten die gerandomiseerd worden naar NOM en een falen van NOM hebben: (re-)embolisatie of heelkunde? wie bepaalt dit?

2.3 Knowledge transfer and implementation

VG	G	S	M	U
	X			

Consider:
- analysis of the context in which implementation is to take place;
- extent to which target groups are mentioned and involved;
- participation of stakeholders, other than target groups;
- prospect of structural incorporation in system;
- the plans for knowledge utilisation.

Please indicate the strong and weak(er) points.

lijkt OK

2.4 Project group and collaboration

VG	G	S	M	U
	X			

Consider:
- relevant expertise and disciplines;
- familiarity with research area;
- prior activities and products.
- collaboration with other institutions (note that within the TopZorg Programme collaboration with either a University Medical Center and/or scientific research institute is obliged)

Please indicate the strong and weak(er) points.

ik ga ervan uit dat de lokale interventieradiologen voldoende kennis en kunde hebben om een SAE correct uit te voeren, mede gezien dit geen alledaagse interventie is.

2.5 Feasibility

VG	G	S	M	U
	X			

Consider:
- prospects of achieving the objective(s) using this strategy;
- realistic phasing and timetable;
- availability of facilities/staff;
- research protocol;
- realistic number of patients/institutes/organisations;
- recruitment of patients. If applicable: is randomisation feasible?

Please indicate the strong and weak(er) points.

recruitment hangt af van aanbod van de deelnemende lokale ziekenhuizen; echter milttrauma is op zich geen alledaagse pathologie

2.6 Overall quality assessment

VG	G	S	M	U
	X			

Give your overall quality assessment regarding this grant application. Please indicate the most important strong and weak(er) points.

ok

3. Budget

Legenda: TH (Too high), R (Realistic), TL (Too low), NJ (No judgement)

3.1 Budget

TH	R	TL	NJ
	X		

Please give your judgement of the budget based on the data supplied in the grant application. There is a comprehensive overview of the requested budget (in Dutch).

If you are not able to assess the requested budget please type 'No Judgement' in the textbox.

ok
